# Clinical profile of an unselected population with heart failure treated with vericiguat in real life: differences with the VICTORIA trial

**DOI:** 10.3389/fcvm.2024.1504427

**Published:** 2025-01-06

**Authors:** Alberto Esteban-Fernández, Alejandro Recio-Mayoral, Raquel López-Vilella, Gregorio de Lara, Moisés Barrantes-Castillo, Inés Gómez-Otero, Julio Nuñez-Villota, Carolina Robles-Gamboa, José López-Aguilera, Ángel Iniesta-Manjavacas, Paula Fluviá, Francisco Pastor-Pérez, Laia Belarte-Tornero, Gonzalo Alonso-Salinas, Pablo Díez-Villanueva

**Affiliations:** ^1^ Cardiology Service, Hospital Universitario Severo Ochoa, Madrid, Spain; ^2^ Faculty of Health Sciences, Alfonso X El Sabio University, Madrid, Spain; ^3^ Cardiology Service, Hospital Universitario Virgen Macarena, Sevilla, Spain; ^4^ Cardiology Service, Hospital Universitari I Politécnic La Fe, Valencia, Spain; ^5^ Centro de Investigación Biomedica en Red Cardiovascular (CIBERCV), Madrid, Spain; ^6^ Cardiology Service, Hospital Universitario de Torrevieja, Alicante, Spain; ^7^ Cardiology Service, Hospital de Palamós, Gerona, Spain; ^8^ Cardiology Service, Hospital Clínico Universitario de Santiago de Compostela, La Coruña, Spain; ^9^ Cardiology Service, Hospital Clínico Universitario de Valencia, Valencia, Spain; ^10^ Insitute of Health Research-INCLIVA, Valencia, Spain; ^11^ Universidad de Valencia, Valencia, Spain; ^12^ Cardiology Service, Hospital Universitario de Toledo, Toledo, Spain; ^13^ Cardiology Service, Hospital Universitario Reina Sofía, Córdoba, Spain; ^14^ Cardiology Service, Hospital Universitario LaPaz, Madrid, Spain; ^15^ Cardiology Service, Hospital Universitario Dr. Trueta, Gerona, Spain; ^16^ Cardiology Service, Hospital Clínico Universitario Virgen de la Arixaca, Murcia, Spain; ^17^ Cardiology Service, Hospital del Mar, Barcelona, Spain; ^18^ Cardiology Service, Hospital Universitario de Navarra, Pamplona, Spain; ^19^ Cardiology Service, Hospital Universitario de La Princesa, Madrid, Spain

**Keywords:** vericiguat, worsening heart failure, guideline-directed medical therapy, VERISEC registry, heart failure reduced ejection fraction

## Abstract

**Introduction:**

Vericiguat, an oral stimulator of soluble guanylate cyclase, reduces cardiovascular mortality and hospitalisations in patients with heart failure (HF) and reduced ejection fraction, as demonstrated in the VICTORIA trial. This study assessed the real-world use of vericiguat.

**Material and methods:**

This cross-sectional, prospective and multicenter registry (VERISEC) included 776 patients from 43 centres in Spain between December 2022 and October 2023. Of these patients, 79.6% were male, with a mean age of 72.4 (SD:8.7) years. Patients in VERISEC were older and had more comorbidities (diabetes, advanced chronic kidney disease) compared to VICTORIA, with 20% having an estimated glomerular filtration rate below 30 ml/min. They also had higher natriuretic peptide levels [NT-proBNP: 3551 (IQR: 1,675.9, 7,054.0)] pg/ml. Most patients (79.8%) started vericiguat after HF decompensation within the previous three months, with high use of loop diuretics (with an average dose of 65 mg/day) and implanted devices (50%). Sixty percent of patients were on quadruple therapy, with a higher use of sodium-glucose co-transporter 2 inhibitors compared to the VICTORIA trial. Despite the more severe disease in the VERISEC cohort, the implementation of guideline-directed medical therapy was greater than in VICTORIA, although vericiguat was initiated at lower blood pressure levels.

**Conclusions:**

Patients in the VERISEC registry had more severe illness and higher comorbidities compared to those in the VICTORIA, despite receiving optimised treatments. Further research is needed to identify which patients may benefit the most from vericiguat treatment.

## Introduction

Vericiguat, an oral stimulator of soluble guanylate cyclase, enhances the cyclic guanosine monophosphate (GMP) pathway by directly activating soluble guanylate cyclase through a binding site that functions independently of nitric oxide. It also increases the enzyme's sensitivity to endogenous nitric oxide by stabilising its binding to the active site (1, 2). In the VICTORIA trial, vericiguat reduced cardiovascular (CV) mortality and heart failure (HF) hospitalisations in high-risk patients with HF and reduced ejection fraction (HFrEF) when added to optimal medical therapy compared to placebo (3).

Based on this evidence, the ESC clinical practice guidelines recommend that vericiguat may be considered in HFrEF patients with New York Heart Association (NYHA) functional class II-IV who have experienced worsening HF (WHF) despite treatment with a renin-angiotensin-aldosterone system inhibitor (RAASi), a beta-blocker (BB), and a mineralocorticoid receptor antagonist (MRA), with a recommendation IIb (4).

Vericiguat is emerging as a new guideline-directed medical therapy (GDMT) component for patients with WHF. It has a favourable safety profile, including a low risk of arterial hypotension, hyperkaliemia and worsening kidney function. However, the clinical characteristics of patients enrolled in clinical trials often differ from daily clinical practice. This study aimed to describe the characteristics of a real-world, non-selected cohort of patients with HF receiving treatment and compare it with those included in the VICTORIA trial.

## Material and methods

This is a cross-sectional and multicenter national registry (VERISEC) that prospectively includes all consecutive patients who initiated vericiguat in clinical practice across 43 centres in Spain between December 2022 (the date of commercialisation in Spain) and October 2023, according to HF clinical guidelines and medical criteria (4). Baseline clinical and complementary variables included: (a) previous medical history, electrocardiogram, and echocardiographic parameters; (b) medical treatment and doses (percentage of maximum recommended doses according to current recommendations (4), including RAASi, BB, MRA, sodium-glucose co-transporter 2 inhibitors (SGLT2i), and diuretics; (c) implantable cardioverter defibrillator (ICD)/cardiac resynchronisation therapy (CRT); (d) hospital admissions/emergency department visits in the previous 12 months; (e) laboratory parameters: renal function, haemoglobin, electrolytes, and natriuretic peptides. This study complies with the Declaration of Helsinki and was approved by the Ethics Committee, with informed consent obtained from all participants.

Quantitative variables are presented as mean and standard deviation, while categorical data are expressed as frequencies and percentages. Data from patients in the VERISEC registry were compared with those enrolled in the VICTORIA, including all patients who had undergone valid randomisation, by generating *ad hoc* random number distributions based on the variables provided in the original trial. Proportions between the VERISEC and the theoretical population from the VICTORIA were compared using a normal approximation with the z-test. A significance level of 0.05 (two-tailed) was set for all statistical tests. The normality of quantitative variables was assessed using the Kolmogorov-Smirnov test, and comparisons were performed using the Student's *t*-test. All analyses were conducted using STATA software (version 17.0).

## Results

We included 776 patients (79.6% male) with a mean age of 72.4 years (SD: 8.7) (Table 1). Most patients were enrolled in the outpatient HF unit (85.8%), while 14.2% were included during hospitalisation or in the emergency department. Compared with patients included in the VICTORIA trial, those included in our study were older, with a worse baseline clinical profile, including a higher prevalence of diabetes and advanced chronic kidney disease (CKD), though a lower rate of ischemic heart disease. The most frequent etiology in our cohort was ischemic (49.6%), and AF was present in 49.6% of patients.

**Table 1:** Clinical characteristics of VERISEC patients compared to VICTORIA patients.

Characteristics	VERISEC (*N* = 776)	VICTORIA (*N* = 5,050)	*p*
Clinical characteristics			
Age (years)	72.4 (8.7)	67.3 (12.2)	0.00001
Sex (male)-*n* (%)	618 (79.6)	3,842 (76.1)	0.02
Arterial hypertension-*n* (%)	587 (75.6)	3,995 (79.1)	0.01
Diabetes-*n* (%)	411 (53.0)	2,369 (46.9)	0.0007
Dyslipidemia-*n* (%)	540 (69.6)	NA	
BMI (kg/m^2^)	27.7 (5.7)	27.8 (5.9)	0.00001
AF/flutter-*n* (%)	385 (49.6)	2,660 (52.7)	0.09
COPD-*n* (%)	120 (15.5)	867 (17.2)	0.200
Other lung diseases-*n* (%)	116 (14.9)	NA	
HF etiology-*n* (%)			
Coronary artery cardiomyopathy-	385 (49.6)	2,944 (58.3)	0.00001
Dilated cardiomyopathy	226 (29.2)	NA	
Valvular Valvular heart disease	52 (6.7)	NA	
Toxic cardiomyopathy	29 (3.7)	NA	
Other etiologies	84 (10.8)	NA	
Echocardiogram characteristics			
LVEF (%)	30.1 (7.7)	28.9 (8.3)	0.00001
RV dysfunction-*n* (%)	208 (26.8)	NA	
Moderate or severe MR-*n* (%)	209 (26.9)	NA	
Moderate or severe TR-*n* (%)	124 (16.0)	NA	
Moderate or severe pulmonary hypertension-*n* (%)	208 (26.8)	NA	
Functional class-*n* (%)			
NYHA I	25 (3.2)	2 (0.1)	0.00001
NYHA II	447 (57.6)	2,975 (59.0)	0.429
NYHA III	291 (37.5)	2,003 (39.7)	0.210
NYHA IV	11 (1.4)	66 (1.3)	0.773
Index episode			
HF decompensation in previous 3 months^a^	619 (79.8)	4,179 (82.8)	0.03
Hospitalisation for HF in previous 3–6 months	157 (20.4)	871 (17.2)	0.03
Mean number of HF decompensation treated with iv. diuretic in previous 12 months	1.4 (1.1)	NA	
Mean number of HF decompensation treated with an oral diuretic in the previous 12 months	0.6 (0.9)	NA	
Mean number of CV hospitalisation in previous 12 months	1.0 (1.4)	NA	
Mean number of Non-CV hospitalisation in previous 12 months	0.5 (1.0)	NA	

AF, atrial fibrillation; BMI, body mass index; COPD, chronic obstructive pulmonary disease; CV, cardiovascular; HF, heart failure; IV, Intravenous; LVEF, left ventricular ejection fraction; MR, mitral regurgitation; NA, not assessed; NYHA, New York Heart Association; RV, right ventricle; TR, tricuspid regurgitation.

^a^ Included Intravenous diuretic for heart failure with and without hospitalisation in previous 3 months.

Left ventricular ejection fraction (LVEF) and functional class were comparable in both studies, with up to 25% of patients exhibiting biventricular systolic dysfunction and significant valvular heart disease in our cohort. Most patients in our cohort were in NYHA functional class II (57.6%) and III (37.5%). However, patients in our study showed significantly higher levels of natriuretic peptides [median NTproBNP 3551.0 pg/ml (1,675.9, 7,054.0)]. Furthermore, patients in VERISEC carried more often an ICD or a CRT (44.5%).

Most patients in VERISEC (79.8%) started vericiguat following HF decompensation within the previous three months. However, a greater number of patients experienced a decompensation 3–6 months after initiating the medication (20.4%). Before starting vericiguat, VERISEC patients had lower blood pressure values (Table 2) and less frequent treatment with RAASi and BB than in VICTORIA. Conversely, the rate of MRA use was higher than in the VICTORIA. Additionally, the prevalence of triple therapy was lower in VERISEC, in part due to the higher usage of SGLT2i, not considered in VICTORIA, with a high rate of quadruple therapy. A significant proportion of VERISEC patients received high doses of loop diuretics, with many in advanced stages requiring outpatient intermittent administration of levosimendan.

**Table 2:** Medical treatment and different parameters before starting verciguat.

Characteristics	VERISEC (*N* = 776)	VICTORIA (*N* = 5,050)	*p*
Clinical parameters			
SPB (mmHg)	117.1 (18.0)	121.4 (15.7)	0.00001
DBP (mmHg)	69.1 (12.3)	72.8 (11.0)	0.00001
HR (bpm)	71.2 (12.6)	73.1 (13.0)	0.00001
Blood test			
Hemoglobin (mg/dl)	13.7 (2.0)	13.4 (1.9)	0.00001
Creatinine (mg/dl)	1.5 (0.6)	NA	
eGFR (ml/min)	52.1 (22.7)	61.5 (27.2)	0.00001
eGFR<30 ml/min-*n* (%)	149 (19.2)	506 (10.2)	0.00001
Na (mEq/L)	140.0 (3.1)	139.9 (3.4)	0.218
K (mEq/L)	4.4 (0.6)	4.5 (0.5)	0.009
Median NTproBNP (pg/ml)	3,551.0 (1,675.9,7,054.0)	2,816.0 (1,556.0, 5,314.0)	0.00001
ICD/TRC-n (%)	345 (44.5)	1,616 (32.0)	0.00001
Medical treatment			
RAASi-*n* (%)	648 (83.5)	4,431 (87.7)	0.0004
*50% target dose-n (%)*	172 (26.5)	NA	
*100% target dose -n (%)*	178 (27.4)	NA	
BB-*n* (%)	702 (90.5)	4,691 (93.1)	0.004
*50% target dose -n (%)*	211 (29.9)	NA	
*100% target dose -n (%)*	188 (26.6)	NA	
MRA-*n* (%)	606 (78.1)	3,545 (70.3)	0.00001
*50% target dose -n (%)*	298 (49.1)	NA	
*100% target dose -n (%)*	180 (29.7)	NA	
SGLT2i-*n* (%)	712 (91.8)	NA	
Quadruple therapy	461 (59.41)	NA	
Triple therapy	221 (28.5)	3,009 (59.7)	0.00001
Loop diuretics-*n* (%)	683 (88.2)	NA	
*Mean dose (mg)*	65.4 (43.9)	NA	
Nitrates-*n* (%)	63 (8.1)	NA	
PDE5i-*n* (%)	4 (0.5)	NA	
Intermitent Levosimendan-*n* (%)	123 (15.9)	NA	

BB, beta-blocker; CRT, cardiac resynchronisation therapy; DBP, diastolic blood pressure; eGFR, estimated glomerular filtration rate; HR, heart rate; ICD, implantable cardioverter-defibrillator; MRA, mineralocorticoid receptor antagonists; NA, not assessed; NTproBNP, N-terminal prohormone of brain natriuretic peptide; PDE5i, phosphodiesterase-5 inhibitor; RAASi, renin-angiotensin-aldosterone system inhibitors; SBP, systolic blood pressure; SGLT2i, sodium-glucose co-transporter 2 inhibitors.

## Discussion

Current findings thoroughly describe the profile of patients that initiate vericiguat in Spanish real-life settings. These patients displayed more advanced disease facts than those in the VICTORIA trial. Indeed, in the VERISEC registry, patients were older, had greater comorbidity burden, different HF etiology, lower systolic blood pressure, and higher natriuretic peptide levels. Despite the signal of more severe disease, patients in the VERISEC registry have a higher implementation of medical treatment and carried more than reported in the VICTORIA trial.

Previous studies have indicated that nearly half of patients with HFrEF may be eligible for vericiguat, highlighting their high risk of morbidity and mortality (5, 6). Our study's median NT-proBNP level was approximately 3,500, notably higher than those reported in the VICTORIA trial and other HF studies (3). This elevated biomarker, along with the history of recent hospital readmissions, reflects the high-risk profile of the patients, most of whom were treated with loop diuretics at an average dose of 65 mg per day. The use of loop diuretics is consistent with findings from the TIDY-HF registry, where up to 73% of newly diagnosed HFrEF patients received such treatment (7). While the proportion of patients on loop diuretics in VICTORIA is unknown, a German registry of 2,916 patients on vericiguat reported that 77% also received loop diuretics (8).

The underuse of GDMT in patients with WHF has been a persistent challenge, likely due to the difficulty of optimising medical therapy in this population. In our study, vericiguat was initiated at lower blood pressure levels than those in the VICTORIA trial, although other registries reported similar findings (9). Importantly, the efficacy of vericiguat has been demonstrated regardless of baseline blood pressure (10). In addition, a significant proportion of our cohort had CKD, with nearly 20% having an eGFR below 30 ml/min. A subanalysis of VICTORIA, which included fewer patients with advanced CKD, confirmed that vericiguat's benefits are independent of renal function (11).

Another notable finding is the higher rate of device use in our cohort since nearly half of the patients had an implanted device, a proportion that exceeds the rates reported in VICTORIA and other registers (12). Furthermore, the baseline treatment in our cohort was more optimised, with higher utilisation of SGLT2i and a considerable proportion of patients on quadruple therapy. By contrast, the KorAHF registry reported that using BB and MRA was below 60% (9). Similarly, Okami et al., in a registry of 829 patients, found that although more patients were on GDMT, the rates of RAASi and BB use were below 80%, while SGLT2i and MRAs were prescribed to around 50% (12). Other studies have reported similar low treatment rates, although they often lack information on target doses (8). A VICTORIA subanalysis focused on guideline medical therapy adherence found dose-corrected rates of 50.9% for RAASi, 45.4% for BB, and 82.2% for MRA (13). While these doses appear higher than in our study, VICTORIA did not report target doses relative to the maximum recommended by guidelines, possibly reflecting challenges related to contraindications or intolerances. However, therapeutic inertia, often mistaken for contraindications, should be addressed. In our study, nearly 60% of patients were on quadruple therapy. By comparison, 60% of patients in VICTORIA were on triple therapy, and other studies have shown even worse outcomes, with only 27% of HFrEF and WHF patients on triple therapy and 51% on dual therapy (6). Although vericiguat offers a new therapeutic option for WHF patients, achieving GDMT at target doses should remain a primary goal. Vericiguat should be considered an adjunctive therapy or used in cases where there are contraindications to starting or up-titrating other medications (14).

Our study has some limitations. First, it is a real-life study with the limitations inherent to these studies. In addition, given the study's cross-sectional nature, only the patients’ baseline data are available, so it is impossible to evaluate the events and tolerance of the treatment during follow-up. Future studies should identify the patient profiles that benefit the most from vericiguat. The ongoing VICTOR trial (NCT05093933) is expected to provide insight into the efficacy of vericiguat in lower-risk patients, particularly those without recent HF decompensation. However, the use of vericiguat in more advanced patients—16% of our cohort were on intermittent inotropic therapy (15), possibly reflecting very high natriuretic peptide levels—may not be appropriate (16).

In conclusion, this study highlights the potential use of vericiguat in high-risk HFrEF patients post-WHF, particularly those with low blood pressure and CKD. While vericiguat demonstrates benefits across a range of baseline characteristics, optimising GDMT remains crucial. Further research is needed to define better the patient populations that derive the most benefit from vericiguat and to explore its role in less severe cases of HF.

## Data Availability

The raw data supporting the conclusions of this article will be made available by the authors, without undue reservation.

## VERISEC Registry Researchers

List of contributors:

Mikel Taibo, Esther Sánchez Corral, Antonio García Quintana, Mario Galván, José M, García Pinilla, Ainoha Robles, Francisco Bermúdez, Silvia López, Pedro Pájaro, Lorenzo Fácila, Sonia Mirabet, Clara Simón, Luis Almenar, Juan Gorriz, David González Calle, Pau Codina, Jara Gayán, Vanessa Escolar, Ainara Lozano, Carrillo Alemán, Cristina Goena, Marta Jiménez, Marta Cobo, Juan Carlos López Azor, Sara Corredera, Pedro Caravaca, María Melendo, Dina Nieto, Julia Seller, Carolina Ortiz, María Thiscal López, José María Viétez Flórez, Juan Luis Bonilla, Antonia Pomares, Aleix Olivella, Francisco Camacho, Beatriz Calvo, Montserrat Cardona and Ricardo Martínez-González.
